# Effect of Immunotherapy on Late Elderly Patients With Unresectable Hepatocellular Carcinoma: A Real‐World Clinical Study

**DOI:** 10.1002/cam4.71171

**Published:** 2025-08-26

**Authors:** Shun Kaneko, Yasuhiro Asahina, Miyako Murakawa, Shunsuke Ueyama, Hideki Watanabe, Chiaki Maeyashiki, Akiko Kusano‐Kitazume, Ayako Sato, Kozue Uchidate, Taro Watabe, Takehito Asakawa, Sho Watanabe, Junko Fujiki, Isamu Shibata, Shinya Oooka, Hitoshi Kurata, Mao Tsuchiya, Takashi Fujii, Keiichi Akahoshi, Daisuke Ban, Kento Inada, Tsubasa Nobusawa, Tomohiro Mochida, Keiya Watakabe, Taro Shimizu, Jun Tsuchiya, Masato Miyoshi, Fukiko Kawai‐Kitahata, Sayuri Nitta, Mina Nakagawa, Sei Kakinuma, Ryuichi Okamoto, Ochanomizu Liver Conference Study Group

**Affiliations:** ^1^ Department of Gastroenterology and Hepatology Institute of Science Tokyo Tokyo Japan; ^2^ Department of Gastroenterology and Hepatology Tsuchiura Kyodo General Hospital Ibaraki Japan; ^3^ Department of Gastroenterology and Hepatology Yokosuka Kyosai Hospital Kanagawa Japan; ^4^ Department of Gastroenterology and Hepatology Musashino Red Cross Hospital Tokyo Japan; ^5^ Department of Gastroenterology and Hepatology Tokyo Metropolitan Tama Medical Center Tokyo Japan; ^6^ Department of Gastroenterology and Hepatology Tokyo Metropolitan Bokutoh Hospital Tokyo Japan; ^7^ Department of Gastroenterology and Hepatology JA Toride Medical Center Ibaraki Japan; ^8^ Department of Gastroenterology and Hepatology Ome Medical Center Tokyo Japan; ^9^ Department of Gastroenterology and Hepatology Yokohama City Minato Red Cross Hospital Kanagawa Japan; ^10^ Department of Gastroenterology and Hepatology Soka Municipal Hospital Saitama Japan; ^11^ Department of Gastroenterology and Hepatology Kashiwa Municipal Hospital Chiba Japan; ^12^ Department of Gastroenterology National Hospital Organization Disaster Medical Center Tokyo Japan; ^13^ Department of Medical Oncology Showa General Hospital Tokyo Japan; ^14^ Department of Gastroenterology and Hepatology Tokyo Metropolitan Ohtsuka Hospital Tokyo Japan; ^15^ Department of Gastroenterology and Hepatology Tokyo Kyosai Hospital Tokyo Japan; ^16^ Department of Gastroenterology and Hepatology Tokyo Metropolitan Hiroo Hospital Tokyo Japan; ^17^ Department of Hepatobiliary and Pancreatic Surgery Institute of Science Tokyo Tokyo Japan; ^18^ Center for Healthcare Education Institute of Science Tokyo Tokyo Japan; ^19^ Department of Clinical and Diagnostic Laboratory Science Institute of Science Tokyo Tokyo Japan

**Keywords:** ALBI score, atezolizumab plus bevacizumab, durvalumab‐tremelimumab, hepatocellular carcinoma, post‐ICI treatment

## Abstract

**Background/Aim:**

The global aging population includes an increasing number of elderly patients with hepatocellular carcinoma (HCC). This study aimed to clarify the real‐world outcomes, prognostic factors, and appropriate administration indicators for immunotherapy in elderly HCC patients.

**Methods:**

This retrospective multicenter study analyzed 286 patients with unresectable HCC who received first‐line immunotherapy (atezolizumab–bevacizumab or durvalumab‐tremelimumab) between November 2020 and January 2024. Patients were categorized into the late elderly (LE; ≥ 75 years, *n* = 117) and non‐late elderly (non‐LE; ≤ 74 years, *n* = 169) groups. Baseline characteristics, overall survival (OS), progression‐free survival (PFS), and prognostic factors were evaluated.

**Results:**

The LE group had significantly poorer performance status, lower albumin–bilirubin (ALBI) scores, lower alpha‐fetoprotein (AFP) and alanine transaminase levels, higher creatinine levels, and were significantly less likely to receive post‐immune checkpoint inhibitor (ICI) treatment compared with the non‐LE group (56.2% vs. 38.4%, *p* = 0.0038). Median OS and PFS for the LE group were 25.6 and 10.5 months, respectively. The LE group demonstrated a comparable disease control rate (82.0%) and safety profile. The ALBI score was a significant prognostic factor for both groups. Post‐ICI treatment significantly improved OS only in the non‐LE group, even after propensity score matching for ALBI score and AFP levels.

**Conclusions:**

Immunotherapy is effective and well‐tolerated in LE patients with unresectable HCC, particularly in those with preserved liver function (mALBI grade 1/2a). Post‐ICI treatment significantly benefits non‐LE patients, with limited impact on LE patients, highlighting the need for therapeutic strategies based on age and liver function.

## Graphical abstract

Immunotherapy demonstrates efficacy and tolerability in late elderly (≥ 75 years) patients with unresectable hepatocellular carcinoma, especially those with preserved liver function (mALBI 1/2a). Post‐immunotherapy treatment provides additional survival benefit mainly in non‐late elderly (≤ 74 years). Age and liver function should guide personalized therapeutic strategies for unresectable hepatocellular carcinoma.

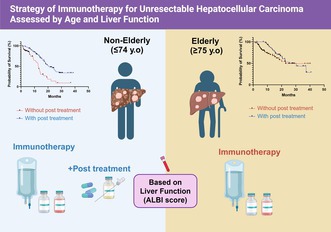

**Abbreviations**
AB: atezolizumab and bevacizumab
AE: adverse event
AFP: alpha‐fetoprotein
ALBI: albumin–bilirubin
ALT: alanine aminotransferase
CI: confidence interval
CP: Child–Pugh
CRP: C‐reactive protein
CTLA‐4: cytotoxic T lymphocyte‐associated protein 4
DCR: disease control rate
DT: durvalumab and tremelimumab
eGFR: estimated glomerular filtration rate
HBV: hepatitis B virus
HCC: hepatocellular carcinoma
HCV: hepatitis C virus
HR: hazard ratio
ICI: immune checkpoint inhibitor
irAEs: immune‐related AEs
LE: late elderly
mALBI: modified albumin–bilirubin
ORR: objective response rate
OS: overall survival
PD‐L1: programmed cell death 1‐ ligand 1
PFS: progression‐free survival
PS: performance status
PSM: propensity‐score matched
RECIST: response evaluation criteria in solid tumors
TKIs: tyrosine kinase inhibitors
Tregs: regulatory T Cells
UPCR: urinary protein‐to‐creatinine ratio

## 1 Introduction

Aging is increasing worldwide. In 2021, the global population aged > 60 years accounted for 13.7%. The older population is predicted to reach 19.2% by 2040. In 2020, of the 19.3 million new cancer cases worldwide, 64% occurred in individuals aged ≥ 60 years, contributing to 71.3% of cancer‐related deaths [1]. All countries face major challenges in ensuring that their health and social systems are ready to make the most of this demographic shift [2].

Liver cancer is a global threat, with 905,700 new diagnoses and 830,200 fatalities reported globally in 2020. The number of new cases and deaths from liver cancer could rise by > 55% by 2040 [3]. From 2005 to 2019, the incidence of liver cancer in people aged ≥ 60 years significantly increased [1]. Hepatocellular carcinoma (HCC) is a leading cancer, accounting for 90% of liver cancer cases [4, 5]. The aging trend among patients with HCC has been observed, particularly in Japan [6, 7]. Age is a risk factor for HCC development [8, 9]. Advances in viral hepatitis control, such as the use of nucleos(t)ide analogs for chronic hepatitis B [10, 11] and direct‐acting antivirals for chronic hepatitis C [12], have shifted the primary etiologies of HCC. As a result, HCC is now being diagnosed more frequently in late elderly (LE) patients with relatively preserved liver function [13]. Biological changes such as declines in muscle mass and function have been reported in individuals aged 75 and older [14], and previous studies have evaluated health status and functional capacity using this age threshold [15]. In Japan, turning 75 marks a transition to a different public insurance system, suggesting a practical basis for recognizing this age group. Moreover, international trends in the classification of older adults are increasingly shifting toward using 75 years as a key reference point. Therefore, we defined LE as patients aged ≥ 75 years in this study.

In addition, systemic therapy for advanced HCC has remarkably progressed. The combination therapy of atezolizumab (an anti‐PD‐L1 immune checkpoint inhibitor [ICI]) and bevacizumab [16], or the combination of durvalumab (an anti‐PD‐L1 ICI) and tremelimumab (an anticytotoxic T lymphocyte‐associated protein 4 (CTLA‐4) antibody) [17], has become available.

In general, clinical trials for systemic therapy often include a low number of elderly patients [18]. A meta‐analysis of 17 randomized control trials involving patients treated with nivolumab, pembrolizumab, or atezolizumab for metastatic solid tumors showed that patients aged ≥ 65 years had similar overall survival (OS) and progression‐free survival (PFS) to those aged < 65 years old [19]. However, aging is associated with immunosenescence. In HCC, changes in the tumor microenvironment during immunosenescence affect antigen recognition and presentation by antigen‐presenting cells. In addition, increased counts of cells such as myeloid‐derived suppressor cells and regulatory T cells (Tregs), along with other related molecules, interfere with the action of cytotoxic T lymphocytes. This immunosenescence has raised concerns that immunotherapy may not be as effective in elderly patients [20]. Conversely, a melanoma mouse model demonstrated that older mice exhibited a greater response to anti‐PD1 therapy than younger mice, particularly with the blockade of Tregs. This indicates that a decrease in Tregs with aging may enhance immunotherapy response [21]. However, many uncertainties remain, and further evaluation of its clinical utility is needed. Few studies have reported on the efficacy and safety of immunotherapy in the LE (aged > 75 years) that reflect the real‐world population distribution. Thus, detailed reports on indicators and prognostic factors, including post‐ICI treatment outcomes for elderly patients with unresectable HCC in the immunotherapy era, are needed. This study aimed to reveal the actual conditions of immunotherapy, prognostic factors, and indicators in LE patients with unresectable HCC.

## 2 Methods

### 2.1 Patients

This retrospective study enrolled 339 patients who received atezolizumab and bevacizumab (AB) or durvalumab and tremelimumab (DT) therapy as first‐line immunotherapy from November 2020 to January 2024 at 16 hospitals. Of these patients, 53 were excluded because of crossover treatment (not receiving first‐line immunotherapy), short follow‐up periods (< 1 month), and/or missing data on blood tests and treatment. Finally, 286 patients (including 242 patients aged ≥ 65 years) were included. HCC was diagnosed based on either pathological confirmation or radiological findings, such as typical tumor arterial enhancement, followed by a washout pattern in the portal venous or equilibrium phase on dynamic computed tomography or magnetic resonance imaging, following practice guidelines [22, 23, 24]. Baseline characteristics and prognosis were analyzed by dividing the patients into two age‐based groups: LE (age ≥ 75 years) and non‐LE (age ≤ 74 years). Liver function was assessed using the Child–Pugh (C–P) classification, albumin–bilirubin (ALBI), and modified ALBI (mALBI) grades. The ALBI score was calculated as previously reported [25]. Based on the calculated score, ALBI was classified as grade 1, ≤ −2.60; grade 2, > −2.60 to ≤ −1.39; and grade 3, > −1.39. Using a previously reported cutoff ALBI score of −2.270, ALBI grade 2 was further divided into subgrades 2a and 2b. The four ALBI grades were named as mALBI grade [26]. The best radiologic response was evaluated according to the Response Evaluation Criteria in Solid Tumors (RECIST) 1.1 and the modified RECIST as previously reported [27, 28, 29].

This study was approved by the ethics committee of the Institute of Science Tokyo Hospital (Confirmation no. M2018‐223) and was conducted following the 2013 revision of the Declaration of Helsinki.

### 2.2 Systemic Therapy (Immunotherapy)

Atezolizumab (1200 mg) and bevacizumab (15 mg/kg body weight) were administered intravenously every 3 weeks. Tremelimumab, at a dose of 300 mg for one cycle, and durvalumab, at 1500 mg/body, were administered intravenously every 4 weeks. Treatment was discontinued in the case of any unacceptable or serious adverse event (AE) or clinical tumor progression. AEs were assessed according to the National Cancer Institute common terminology criteria for adverse events version 5.0. AB or DT regimen was temporarily interrupted when a patient developed any grade 3, any unacceptable grade 2, or drug‐related AE and was resumed when the symptoms improved to grade < 2.

### 2.3 Study Endpoint

The study endpoint was the OS, which was calculated from the start of AB or DT administration as first‐line immunotherapy until death by any cause or the last follow‐up. The baseline factors associated with OS were analyzed.

Prognostic factors included age, viral hepatitis etiology (hepatitis B virus [HBV] and hepatitis C virus [HCV]), performance status (PS), serum neutrophil‐to‐lymphocyte ratio, ALBI score, alpha‐fetoprotein (AFP), and C‐reactive protein (CRP) levels before immunotherapy; first‐line immunotherapy regimen; proteinuria; immune‐related AEs (irAEs) during immunotherapy; and post‐ICI treatment.

### 2.4 Statistical Analyses

Categorical data were compared using the chi‐square or Fisher's exact tests. Continuous variables were analyzed using the Student t‐test or the Mann–Whitney U test. The Kaplan–Meier method was used to generate the cumulative survival rates, which were compared using the log rank (Mantel–Cox) test. The prognostic factors were analyzed using the Cox proportional hazards model. *p* values < 0.05 were considered significant. GraphPad Prism software (GraphPad Software, San Diego, CA, USA) and EZR (Saitama Medical Center, Jichi Medical University, Shimotsuke, Japan) were used for the statistical analyses.

## 3 Results

### 3.1 Patient Characteristics

The characteristics of the 286 patients who received AB or DT for HCC are presented in Table 1. Among them, 117 (40.9%) were LE patients. The median age was 79 (range, 75–91) years, and 83.8% of the patients were men. The etiologies of the underlying liver disease were HBV (*n* = 8, 6.8%), HCV (*n* = 32, 27.4%), alcohol (*n* = 30, 25.6%), and others (*n* = 47, 40.2%).

**TABLE 1:** Baseline clinical characteristics of patients treated with immunotherapy for hepatocellular carcinoma.

	Non‐LE (≤ 74 y.o)	LE (≥ 75 y.o)	*p*
*n*	169	117	
Age (years), median (range)	68 (34 to 74)	79 (75 to 91)	< 0.001***
Sex: Male (*n*, %)	149 (88.2%)	98 (83.8%)	0.29
BMI	23.3 (15.4 to 41.6)	23.6 (16.0 to 36.3)	0.32
ECOG PS 0/1/2	143/25/1	82/32/3	0.006**
Etiology (viral/non‐viral)			0.176
HBV	30 (17.8%)	8 (6.8%)	
HCV	42 (24.9%)	32 (27.4%)	
Alcohol	53 (31.4%)	30 (25.6%)	
Others	44 (26.1%)	47 (40.2%)	
Child–Pugh class			0.003**
A	133 (78.7%)	106 (90.6%)	
B	36 (21.3%)	11 (9.4%)	
ALBI score	−2.30 (−3.36 to −0.918)	−2.478 (−3.38 to −0.40)	0.01*
mALBI grade 1/2a/2b/3	49/41/72/7	43/38/33/3	
mALBI grade 1/2a	90 (53.2%)	81 (69.2%)	0.00715**
AFP (ng/mL)	37.3 (1.5 to 852,122)	15.1 (1.0 to 375,007)	0.02*
AFP > 100 (ng/mL)	74 (43.7%)	34 (29.1%)	0.0132*
PIVKA‐II (mAU/mL)	373.7 (13 to 2,349,239)	238.5 (13 to 137,317)	0.218
NLR	2.55 (0.7 to 11.1)	2.61 (0.19 to 8.37)	0.897
Cirrhosis (*n*, %)	125 (74.0%)	79 (67.5%)	0.287
ALT (IU/L)	30 (9 to 202)	23 (6 to 158)	0.003**
Creatinine (mg/dL)	0.80 (0.32 to 5.43)	0.84 (0.45 to 3.73)	0.039*
eGFR (mL/min/1.73 m^2^)	71.6 (8.9 to 168.9)	63.44 (13.1 to 100.8)	< 0.001***
Proteinuria			0.585
−	116 (69.5%)	72 (62.6%)	
±	10 (6.0%)	12 (10.4%)	
1+	24 (14.4%)	18 (15.7%)	
2+	12 (7.2%)	9 (7.8%)	
3+	5 (3.0%)	3 (2.6%)	
4+	0 (0%)	1 (0.9%)	
≥ 2+	17 (10.2%)	13 (11.3%)	0.845
CRP (mg/dL)	0.30 (0.02 to 12.8)	0.27 (0.01 to 9.13)	0.126
BCLC stage			0.155
0/A (early stage)	14 (8.3%)	13 (11.2%)	
B (intermediate stage)	87 (51.5%)	71 (60.7%)	
C (advanced stage)	68 (40.2%)	33 (28.2%)	
Treatment line 1st/2nd/3rd/4th/5th/6th	114/42/7/3/2/1	85/19/6/6/0/1	0.21
AB or DT	159/10	109/8	0.807
Post ICI treatment	95 (56.2%)	45 (38.4%)	0.0038**

*Note:* Statistical significance is indicated as follows: *p < 0.05, **p < 0.01, ***p < 0.001.

Abbreviations: AB, atezolizumab and bevacizumab; AFP, Alpha fetoprotein; ALBI, albumin‐bilirubin; ALT, alanine aminotransferase; BCLC, Barcelona Clinic Liver Cancer; BMI, body mass index; CRP, C‐reactive protein; DT, durvalumab + tremelimumab; eGFR, estimated Glomerular Filtration Rate; HBV, hepatitis B virus; HCV, hepatitis C virus; LE, late elderly; mALBI, modified ALBI; NLR, neutrophil‐to‐lymphocyte ratio; PS, performance status; TKI, tyrosine kinase inhibitor.

The C–P score was grade A in 106 (90.6%) patients and grade B in 11 (9.4%). The median ALBI score was −2.478. In terms of the Barcelona Clinic Liver Cancer staging, 71 (60.7%) patients had stage B (intermediate) disease, whereas 33 patients (28.2%) had stage C (advanced) disease. AB and DT therapy were administered to 109 (93.1%) and 8 (6.9%) patients, respectively. In addition, 99 patients (84.6%) had a history of prior treatment, and notably, 33 patients (28.2%) had previously received tyrosine kinase inhibitors (TKIs).

Compared with the non‐LE group (*n* = 169), the LE group had significantly poorer PS, lower ALBI scores, lower AFP and ALT levels, higher creatinine levels, and lower estimated Glomerular Filtration Rate (eGFR). No significant differences in proteinuria were observed between the groups, whether assessed by each qualitative level or using “2+” as a cutoff. The non‐LE group was significantly more likely to receive post‐ICI treatment (56.2% vs. 38.4%, *p* = 0.0038).

### 3.2 Objective Response and Adverse Events

Moreover, the efficacy and safety of immunotherapy in the LE group were investigated without adjusting for background factors. The radiological response assessed by RECIST is shown in Table 2A. Despite the lack of a significant difference in the objective response rate (ORR, *p* = 0.103), a higher disease control rate (DCR, 82.0% vs. 69.8%, *p* = 0.026) was observed in the LE group. Although there were no significant differences in baseline characteristics between patients receiving AB or DT therapy, the subgroup analysis by regimen showed consistent results, particularly with the AB therapy (Table S1).

**TABLE 2:** Efficacy and safety of immunotherapy by age group.

A. Best radiological response assessed RECIST 1.1			
	Non LE (≤ 74 y.o)	LE (≥ 75 y.o)	*p*
CR (*n*, %)	5 (3.0%)	10 (8.5%)	
PR (*n*, %)	34 (20.1%)	28 (23.9%)	
SD (*n*, %)	79 (46.7%)	58 (49.6%)	
PD (*n*, %)	51 (30.2%)	21 (18.0%)	
ORR (%)	23.1%	32.4%	0.103
DCR (%)	69.8%	82.0%	0.026*

B. Adverse event			
AB	Non LE (≤ 74 y.o)	LE (≥ 75 y.o)	*p*
Adverse event	74 (46.5%)	54 (49.5%)	0.709
irAE	26 (16.3%)	18 (16.5%)	0.999
irAE grade ≥ 3	3 (1.8%)	4 (3.6%)	0.447
irLiver injury	5 (3.1%)	4 (3.7%)	0.999
Proteinuria	11 (6.9%)	25 (22.9%)	< 0.001***
Hypertension	8 (5.0%)	8 (7.3%)	0.441
Thyroid dysfunction	3 (1.8%)	1 (0.9%)	0.649
Diarrhea	8 (5.0%)	1 (0.9%)	0.08
Infusion reaction	2 (1.3%)	2 (1.8%)	0.999

DT	Non LE (≤ 74 y.o)	LE (≥ 75 y.o)	*p*
Adverse event	7 (70.0%)	6 (75.0%)	0.999
irAE	5 (50.0%)	6 (75.0%)	0.367
irAE Grade ≥ 3	2 (20.0%)	3 (37.5%)	0.608
irLiver injury	2 (20.0%)	2 (25.0%)	0.999

*Note:* Statistical significance is indicated as follows: *p < 0.05, ***p < 0.001.

Abbreviations: AB, atezolizumab and bevacizumab; CR, complete response; DCR, disease control rate; DT, durvalumab + tremelimumab; irAE, immune‐related adverse events; LE, late elderly; ORR, objective response rate; PD, progressive disease; PR, partial response; SD, stable disease.

AEs, including irAEs, were also evaluated, and no significant differences were found between the LE and non‐LE groups. Adverse events were evaluated for each treatment regimen. Although no significant differences in baseline characteristics were observed between the AB and DT regimens, irAEs occurred more frequently in the DT regimen. Regarding the severity of irAEs, no significant differences were observed between the LE and non‐LE groups, as shown in Table 2B. We also investigated the organ‐ or disease‐specific distribution of irAEs and found no significant differences (Fisher's exact test, *p* = 0.805). In contrast, in the AB regimen, which includes anti‐VEGF agents, the incidence of proteinuria was significantly higher in the LE group than in the non‐LE group (22.9% vs. 6.9%, *p* < 0.001).

### 3.3 OS of the LE Group Treated With Immunotherapy

**FIGURE 1:**
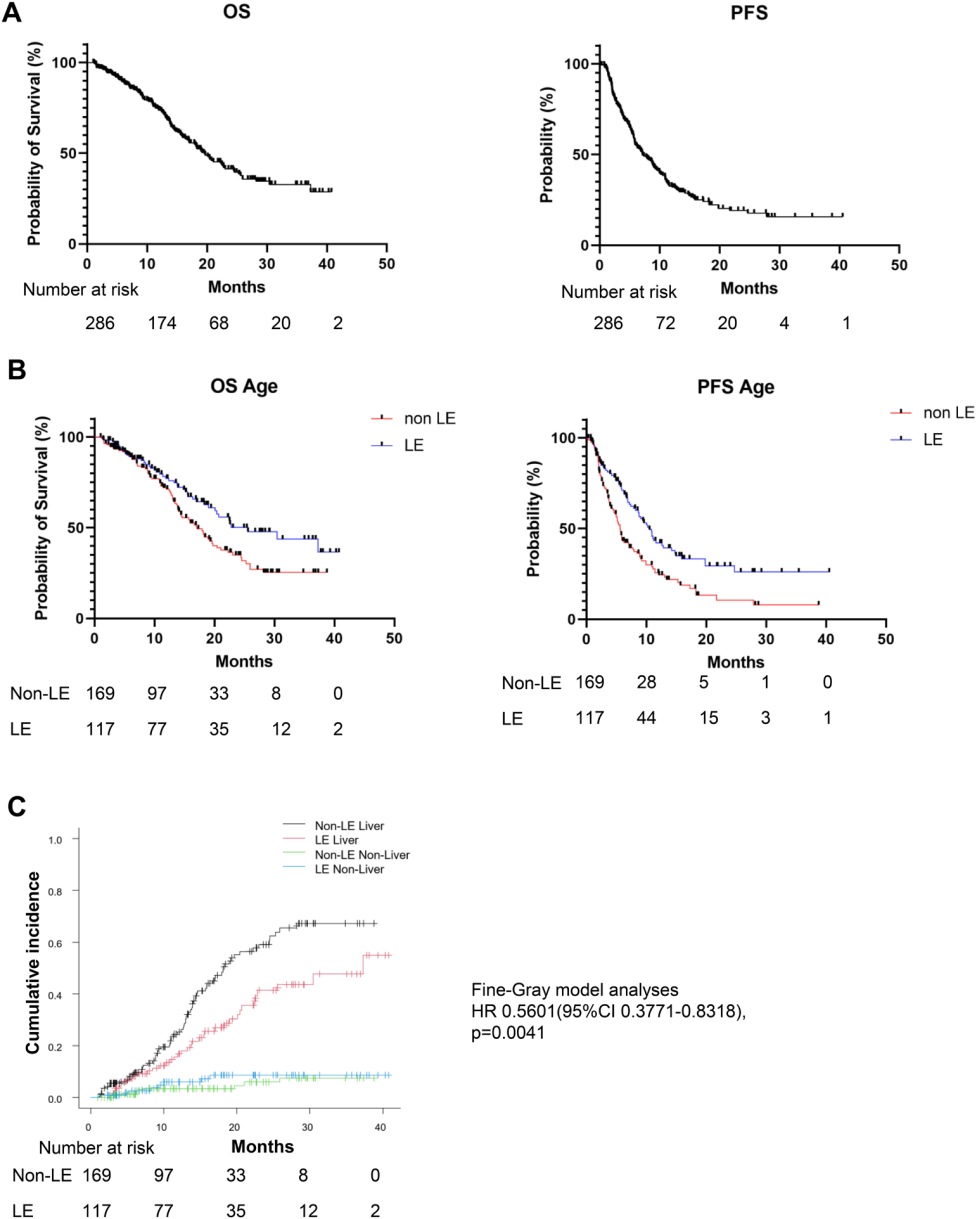
Overall survival (OS) and progression‐free survival (PFS) of patients who received immunotherapy for unresectable hepatocellular carcinoma. (A) OS and PFS. (B) OS and PFS stratified by age group. (C) Liver‐related and non‐liver‐related death events stratified by age group.

The overall median OS and PFS were 19.6 and 7.1 months, respectively (Figure 1A). Survival curves stratified by age (non‐LE and LE groups) are shown in Figure 1B. The median OS and PFS of the LE group, without adjusting for background factors, were 25.6 and 10.5 months, respectively. The subgroup analysis by regimen revealed no significant differences in OS (Figure S1A).

Regarding causes of death, liver‐related deaths occurred in 36 patients in the LE group and 74 patients in the non‐LE group. Non‐liver‐related deaths were observed in 8 patients in both the LE and non‐LE groups (Table S2). Using the Fine–Gray competing risk model, a significant difference in liver‐related deaths was observed between the groups stratified by age (*p* = 0.0041). However, no significant difference was found in other causes of death (Figure 1C).

### 3.4 Prognostic Factors in Patients Treated With Immunotherapy by Age

Univariate and multivariate Cox proportional hazards regression analyses were performed to investigate the prognostic factors for patients treated with immunotherapy. The multivariate analysis identified the ALBI score as an independent prognostic factor in both groups (Table 3):

- LE group: ALBI score (hazard ratio [HR] 1.796, 95% confidence interval [CI] 1.07–3.013, *p* = 0.026).
- Non‐LE group: ALBI score (HR 1.665, 95% CI 1.043–2.657, *p* = 0.032); PIVKA‐II (HR 1.001, 95% CI 1.000–1.002, *p* = 0.002); CRP (HR 1.13, 95% CI 1.039–1.235, *p* = 0.004); and post‐ICI treatment (HR 0.475, 95% CI 0.288–0.783, *p* = 0.003).

**TABLE 3:** Multivariate analyses of factors associated with overall survival of patients treated with ICI.

A. Late elderly (≥ 75 y.o) patients				
	Univariable		Multivariable	
	HR [95% CI]	*p*	HR [95% CI]	*p*
Age	0.9997 (0.9238–1.082)	0.994		
NLR	1.041 (0.8688–1.248)	0.6625		
Viral versus non‐viral	1.371 (0.902–2.084)	0.139		
ALBI score	1.798 (1.072–3.017)	0.0263*	1.796 (1.07–3.013)	0.026*
ECOG PS 0.1.2	1.507 (0.9006–2.521)	0.1185		
AFP (every100 ng/mL)	0.9996 (0.9982–1.001)	0.5416		
PIVKA‐II (every 1000 mAU/mL)	1.005 (0.9878–1.022)	0.584		
CRP	0.9784 (0.7046–1.359)	0.8962		
Proteinuria	0.467 (0.2077–1.05)	0.0656		
irAE	0.7141 (0.3011–1.694)	0.4448		
AB or DT	1.371 (0.1805–10.42)	0.7602		
Post‐ICI treatment	0.7703 (0.4231–1.402)	0.3932		

B. Not late elderly (≤ 74 y.o) patients				
	Univariable		Multivariable	
	HR [95% CI]	*p*	HR [95% CI]	*p*
Age	0.9921 (0.9631–1.022)	0.5995		
NLR	0.9945 (0.975–1.014)	0.584		
Viral versus non‐viral	0.9871 (0.6384–1.526)	0.9535		
ALBI score	2.385 (1.576–3.61)	< 0.001***	1.665 (1.043–2.657)	0.032*
ECOG PS 0.1.2	1.35 (0.7844–2.324)	0.2786		
AFP (every100 ng/mL)	0.999 (0.9994–0.9999)	0.3697		
PIVKA‐II (every 1000 mAU/mL)	1.001 (1.000–1.002)	0.002**	1.001 (1.001–1.002)	< 0.001***
CRP	1.148 (1.055–1.248)	0.001313**	1.13 (1.039–1.235)	0.004**
Proteinuria	0.9175 (0.4224–1.993)	0.8277		
irAE	1.014 (0.55990–1.838)	0.962		
AB or DT	1.185 (0.1586–8.85)	0.866		
Post‐ICI treatment	0.387 (0.2474–0.604)	< 0.001*	0.475 (0.288–0.783)	0.003**

*Note:* Statistical significance is indicated as follows: *p < 0.05, **p < 0.01, ***p < 0.001.

Abbreviations: AB, atezolizumab and bevacizumab; AFP, Alpha fetoprotein; ALBI, albumin‐bilirubin; CI, confidence interval; CRP, C‐reactive protein; DT, durvalumab + tremelimumab; HR, hazards ratios; ICI, Immune Checkpoint Inhibitor; irAE, immune‐related adverse events; NLR, neutrophil‐to‐lymphocyte ratio; PIVKA‐II, protein induced by vitamin K absence or antagonist‐II; PS, performance status.

**FIGURE 2:**
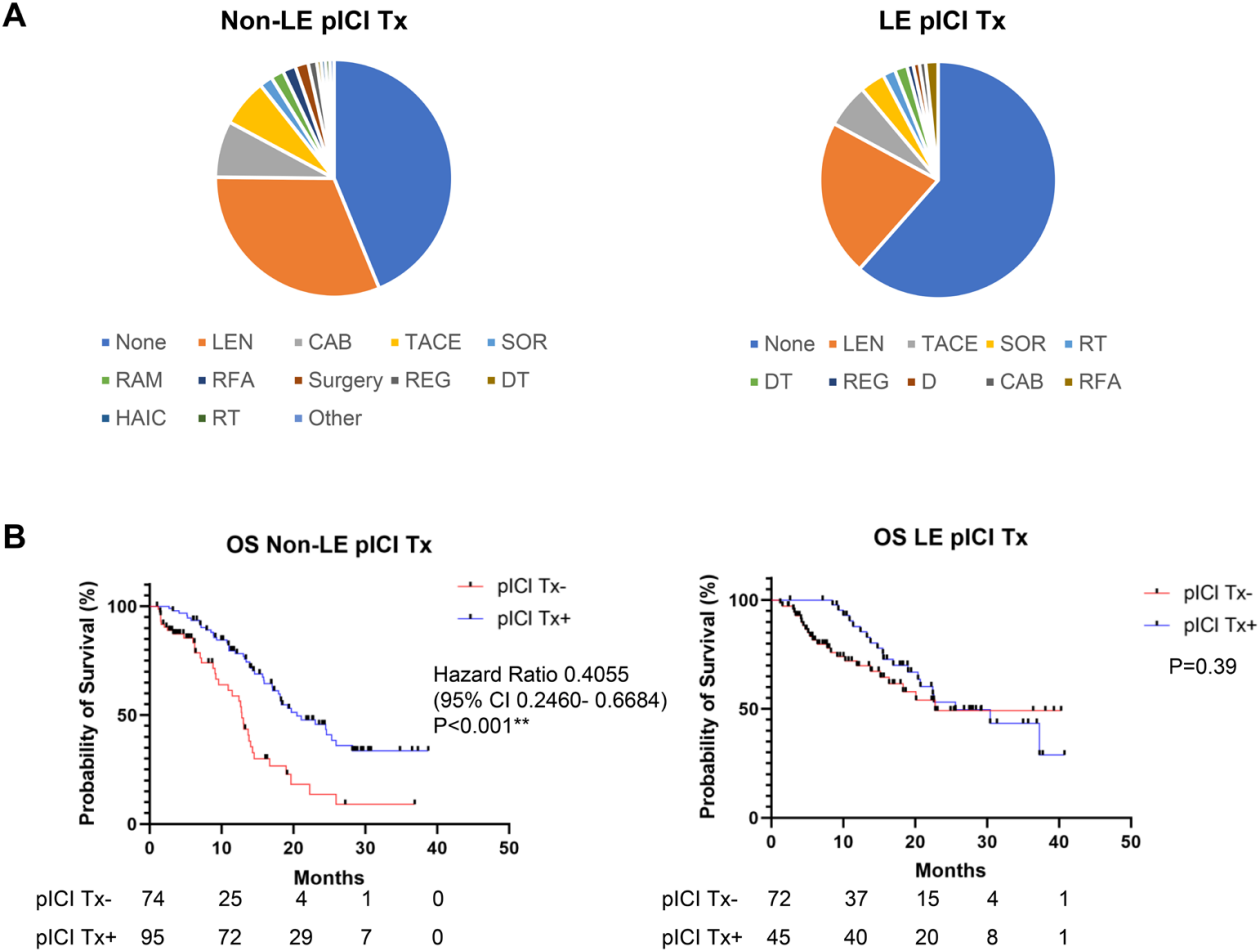
Post‐immune checkpoint inhibitor (ICI) treatments and overall survival. (A) Breakdown of post‐ICI treatments assessed by age. (B) Overall survival according to post‐ICI treatments. CAB, cabozantinib; (D) durvalumab; DT, durvalumab + tremelimumab; HAIC, hepatic arterial infusion chemotherapy; LEN, lenvatinib; RAM, ramcirumab; REG, regorafenib; RFA, radiofrequency ablation; RT, radiation therapy; SOR, sorafenib; TACE, transcatheter arterial chemoembolization.

Further analysis revealed that patients who received post‐ICI treatment had significantly lower ALBI scores in both the non‐LE and LE groups (Table 4). Post‐ICI treatment was more common in the non‐LE group (Table 1). The breakdown of post‐ICI treatment for the non‐LE and LE groups is shown in Figure 2A and Table S3. In the LE group, no significant difference in OS was observed between patients with and without post‐ICI treatment (*p* = 0.39). However, in the non‐LE group, those who received post‐ICI treatment had significantly longer OS (HR 0.405, 95% CI 0.246–0.668, *p* < 0.001, Figure 2B).

**TABLE 4:** Baseline clinical characteristics of patients with or without post immunotherapy treatment.

A. Late elderly (≥ 75 y.o) patients			
	pICI Tx no	pICI Tx yes	*p*
*n*	72	45	
Age (years), median (range)	79.5 (75 to 91)	79 (75 to 90)	0.366
Sex: Male (*n*, %)	58 (80.6%)	40 (88.9%)	0.306
BMI	24.9 (17.3 to 36.3)	22.6 (16.0 to 29.9)	0.026*
ECOG PS 0/1/2	50/19/3	32/13/0	0.514
Etiology (viral/non‐viral)			0.84
HBV	6 (8.3%)	2 (4.4%)	
HCV	18 (25.0%)	14 (31.1%)	
Alcohol	19 (26.4%)	11 (24.4%)	
Others	29 (40.3%)	18 (40.0%)	
Child–Pugh class			0.839
A	64 (88.9%)	42 (93.3%)	
B	8 (11.1%)	3 (6.7%)	
ALBI score	−2.38 (−3.38 to −0.40)	−2.56 (−3.27 to −1.58)	0.005**
mALBI grade 1/2a/2b/3	21/24/24/3	22/14/9/0	0.093
mALBI grade 1/2a	45 (62.5%)	36 (80.0%)	0.0635
AFP (ng/mL)	18.8 (1.0 to 375,007)	6.9 (1.2 to 284,723)	0.05
AFP > 100 (ng/mL)	25 (34.7%)	9 (20.0%)	0.0985
NLR	2.65 (0.19 to 8.37)	2.56 (1.25 to 6.33)	0.792
Cirrhosis (*n*, %)	51 (70.8%)	27 (62.2%)	0.417
ALT (IU/L)	27 (6 to 158)	18 (6 to 123)	0.026*
Creatinine (mg/dL)	0.84 (0.45 to 1.93)	0.84 (0.54 to 1.88)	0.775
CRP (mg/dL)	0.29 (0.01 to 9.13)	0.19 (0.01 to 2.91)	0.183
CRP > 1 (mg/dL)	15 (20.8%)	3 (6.7%)	0.062
BCLC stage			0.787
0/A (early stage)	7 (9.7%)	6 (13.3%)	
B (intermediate stage)	45 (62.5%)	26 (57.8%)	
C (advanced stage)	20 (27.8%)	13 (28.9%)	
Treatment line 1st/2nd/3rd/4th/5th/6th	52/12/3/3/0/1	32/7/3/3/0/0	0.911
AB or DT	65/7	44/1	0.15

B. Not late elderly (≤ 74 y.o) patients			
	pICI Tx no	pICI Tx yes	*p*
*n*	74	95	
Age (years), median (range)	70 (34 to 74)	68 (34 to 74)	0.049*
Sex: Male (*n*, %)	65 (87.8%)	84 (88.4%)	0.999
BMI	23.34 (17.6 to 33.1)	23.1 (15.4 to 41.6)	0.862
ECOG PS 0/1/2	61/12/1	82/13/0	0.512
Etiology (viral/non‐viral)			0.877
HBV	13 (17.6%)	17 (17.9%)	
HCV	18 (24.3%)	24 (25.3%)	
Alcohol	24 (32.4%)	29 (30.5%)	
Others	19 (25.7%)	25 (26.3%)	
Child–Pugh class			< 0.001***
A	48 (64.9%)	85 (89.5%)	
B	26 (35.1%)	10 (10.5%)	
ALBI score	−2.18 (−3.36 to −0.92)	−2.45 (−3.28 to −1.34)	< 0.001***
mALBI grade 1/2a/2b/3	15/17/36/6	34/24/36/1	0.022*
mALBI grade 1/2a	32 (43.2%)	5 (61.0%)	0.0294*
AFP (ng/mL)	14.5 (2.0 to 168,837)	84.9 (1.5 to 852,122.7)	0.112
AFP > 100 (ng/mL)	28 (37.8%)	46 (48.4%)	0.211
NLR	2.84 (0.76 to 13.0)	2.48 (1.09 to 6.33)	0.202
Cirrhosis (*n*, %)	56 (75.7%)	69 (72.6%)	0.725
ALT (IU/L)	27 (9 to 202)	34 (9 to 165)	0.102
Creatinine (mg/dL)	0.78 (0.32 to 2.07)	0.81 (0.50 to 5.43)	0.624
CRP (mg/dL)	0.33 (0.01 to 12.89)	0.25 (0.01 to 10.7)	0.167
BCLC stage			0.597
0/A (early stage)	8 (10.8%)	6 (6.4%)	
B (intermediate stage)	37 (50.0%)	50 (52.6%)	
C (advanced stage)	29 (39.2%)	39 (41.1%)	
Treatment line 1st/2nd/3rd/4th/5th/6th	55/13/4/1/1/0	59/29/3/2/1/1	0.317
AB or DT	66/8	93/2	0.022*

*Note:* Statistical significance is indicated as follows: *p < 0.05, **p < 0.01, ***p < 0.001.

Abbreviations: AB, atezolizumab and bevacizumab; AFP, Alpha fetoprotein; ALBI, albumin‐bilirubin; ALT, alanine aminotransferase; BCLC, Barcelona Clinic Liver Cancer; BMI, body mass index; CRP, C‐reactive protein; DT, durvalumab + tremelimumab; HBV, hepatitis B virus; HCV, hepatitis C virus; LE, late elderly; mALBI, modified ALBI; NLR, neutrophil‐to‐lymphocyte ratio; pICI Tx, post immune check point inhibitor treatment; PS, performance status; TKI, tyrosine kinase inhibitor.

### 3.5 Prognostic Significance of Liver Function and Post‐ICI Treatment in the Non‐LE and LE Groups

**FIGURE 3:**
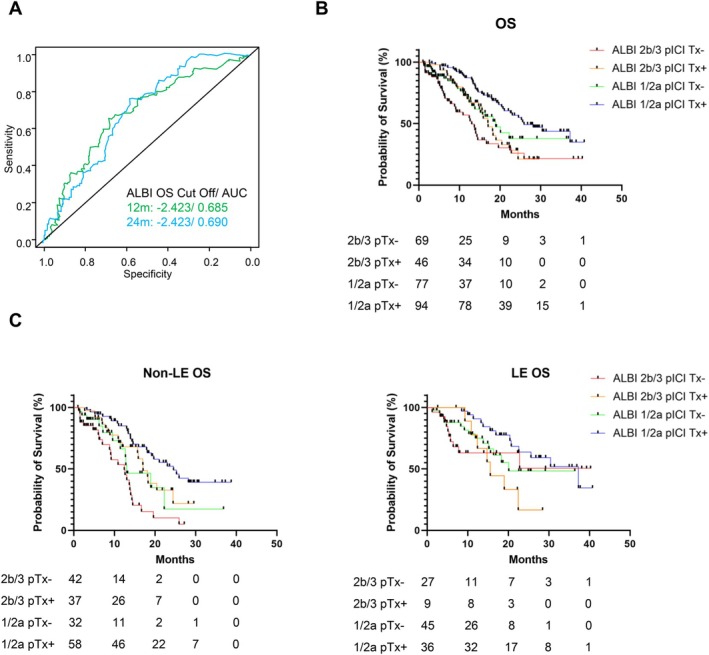
Overall survival stratified by the ALBI score and post‐ICI treatments in patients receiving immunotherapy. (A) Optimal cutoff for the ALBI score in predicting 12‐ and 24‐month survival. (B) Overall survival stratified by mALBI grades and post‐ICI treatments. (C) Overall survival stratified by mALBI grades and post‐ICI treatments assessed by age. ICI, immune checkpoint inhibitor; mALBI, modified albumin–bilirubin.

The ALBI score, a significant prognostic factor common to both the LE and non‐LE groups, was further investigated. The optimal cutoff for the ALBI score in predicting 12‐ and 24‐month OS was −2.423 (area under the curve, 0.685 and 0.690, Figure 3A). Based on this cutoff, the patients were stratified into mALBI grades 1/2a and 2b/3.

For all patients (*n* = 286), OS was significantly stratified by the mALBI grade and post‐ICI treatment (ALBI 2b/3 without pICI Tx, 13.0 months; ALBI 2b/3 with pICI Tx, 17.1 months; ALBI 1/2a without pICI Tx, 18.9 months; and ALBI 1/2a with pICI Tx, 25.9 months; *p* < 0.001, Figure 3B). In the non‐LE group, OS was significantly longer in the ALBI 2b/3 and ALBI 1/2a subgroups with post‐ICI treatment than in those without treatment (ALBI 2b/3 without pICI Tx, 12.0 months; ALBI 2b/3 with pICI Tx, 17.1 months; ALBI 1/2a without pICI Tx, 13.0 months; ALBI 1/2a with pICI Tx, 24.6 months; *p* < 0.001; ALBI 2b/3, *p* = 0.0042; ALBI 1/2a, *p* = 0.0186, Figure 3C left). In the LE group, OS was not significantly stratified by the ALBI grade or post‐ICI treatment (ALBI 2b/3 without pICI Tx, undetermined; ALBI 2b/3 with pICI Tx, 15.5 months; ALBI 1/2a without pICI Tx, 20.1 months; and ALBI 1/2a with pICI Tx, 37.3 months; *p* = 0.17, Figure 3C right). No significant differences in OS were observed in the ALBI 2b/3 (*p* = 0.45) or ALBI 1/2a (*p* = 0.12) subgroups. Furthermore, a total of 221 patients had a history of prior treatment, including 85 who had received TKIs. Associations between prior treatment history or TKI use and OS were evaluated; however, no significant differences in OS were observed between the LE and non‐LE groups in either analysis (Figure S2).

### 3.6 Propensity‐Score Matched (PSM) With the ALBI Score and AFP

To further investigate post‐ICI treatments, PSM was performed, adjusting for ALBI score and AFP, which showed significant differences in the unadjusted background characteristics. The adjusted characteristics of the LE (*n* = 112) and non‐LE (*n* = 112) groups are shown in Table S4.

The median OS was 25.6 months for the LE group and 19.7 for the non‐LE group, with no significant difference (*p* = 0.24, Figure S3A). In the subgroup analyses by post‐ICI treatment and age, no significant survival differences were observed in the LE group with or without post‐ICI treatment (25.6 months vs. undetermined, *p* = 0.41). In contrast, patients in the non‐LE group who received post‐ICI treatment had significantly longer survival than those who did not receive it (23.0 vs. 13.6 months, *p* = 0.0055, Figure S3B). After adjusting for the ALBI score and AFP, post‐ICI treatment was associated with a better prognosis in the non‐LE group; however, no significant prognostic effect was observed in the LE group.

## 4 Discussion

### 4.1 Main Findings

In the clinical practice of the LE group with advanced HCC, immunotherapy has been administered based on evaluations using indicators such as the ALBI score. A survival benefit of approximately 2 years or more was observed regardless of whether post‐ICI treatment following first‐line immunotherapy was administered. Despite the lack of significant differences in the incidence of any AEs or irAEs compared with the non‐LE group, only proteinuria was significantly more frequent in the LE patients. In contrast, for the patients in the non‐LE group with unresectable HCC, post‐ICI treatments contributed to prolonged survival, even in patients who were adjusted by PSM ALBI score and AFP. Therefore, in immunotherapy for the LE group with advanced HCC, it is useful to consider whether liver function is preserved (mALBI grade 1 or 2a) and select first‐line immunotherapy that can be effective and continued without post‐ICI treatment.

### 4.2 Context With the Published Literature

The IMbrave150 trial, a representative study for HCC, included elderly patients. Despite the significantly higher prevalence of comorbidities and complications in this group, a subgroup analysis from the IMbrave150 trial demonstrated improved OS and PFS with AB compared with sorafenib in patients aged ≥ 65 years. These results were consistent with those observed in the overall cohort, and the safety profile in elderly patients was similar. Post hoc analyses also confirmed that AB therapy can be safely used in elderly patients [30].

In real‐world clinical practice, studies have reported similar efficacy of AB therapy in elderly patients. The definition of “elderly” varies, with some studies defining it as ≥ 65 years [31], others as ≥ 75 years with analyses using inverse probability weighting (IPW) [32], and still others compared the efficacy and safety of AB and lenvatinib (LEN) in patients aged ≥ 80 years [33]. Across these studies, the general consensus is that AB therapy is a viable option for elderly patients. However, such reports about DT therapy have not been sufficient.

The significant occurrence of adverse proteinuria events in the LE group requires further examination to determine their impact on prognosis. Previous reports indicate conflicting findings: Yang et al. suggest that proteinuria‐related adverse events during AB therapy are associated with a favorable prognosis [34]; while another study indicates a poor prognosis due to the discontinuation of BEV therapy as a result of proteinuria [35]. Although no definitive conclusion has been reached, Yang et al.'s report explores the mechanism linking baseline low VEGF‐D levels to proteinuria‐related adverse events [34]. In our study, although proteinuria was not significant in the univariate analysis (HR 0.467, 95% CI 0.2077–1.05, *p* = 0.06561) or the multivariate analysis for prognostic factors in the LE group, Urinary Protein‐to‐Creatinine Ratio (UPCR) was available in 143 patients. Among those with both UPCR and qualitative proteinuria data, a significant correlation was observed (correlation coefficient: 0.682; 95% CI: 0.583–0.761; *p* < 0.001, Figure S4A). Based on this correlation, qualitative proteinuria was used for OS analysis; however, baseline qualitative proteinuria was not significantly associated with OS (Figure S4B). Further studies are needed to explore the possibility of proteinuria contributing to favorable prognostic factors.

### 4.3 Strengths and Limitations

This study specifically investigated the relationship among post‐ICI treatment, age groups, and liver function. Various patterns of post‐ICI treatment have been reported. Kawamura et al. categorized post‐ICI treatment into three patterns as part of sequential therapy. The combined use of systemic sequential therapy with > 2 agents and locoregional treatment was defined as multidisciplinary combination therapy, whereas systemic sequential therapy only and repeated locoregional treatment were defined as single treatment procedures [36]. In a previous study about the prognosis of patients with TKI‐treated HCC based on liver function and the extent of portal vein tumor thrombosis, the median OS of patients treated with TKI and additional therapies was significantly longer than that of those treated with TKI only in the ALBI 2b/3 and Vp3/4 groups. The ALBI 1/2a subgroup received TKI therapy for a longer time, regardless of additional therapy [37].

Achieving a deep and durable response in imaging‐based efficacy evaluation is crucial for obtaining a favorable prognosis in HCC management [38, 39]. A notable feature of immunotherapy compared with TKIs is its long‐tail plateau in survival outcomes. As the longest‐term data for immunotherapy in advanced HCC, DT therapy showed a 25.2% 4‐year survival rate, reflecting favorable outcomes [40].

In other cancers, therapies involving CTLA‐4 or PD‐L1 inhibitors have demonstrated that Eastern Cooperative Oncology Group PS 2 is associated with poor prognosis [41]. However, in our cohort, which included patients with PS scores of up to 2, most patients had PS scores of 0 or 1. Although the LE group had significantly lower PS scores, this did not affect the prognosis. The tolerability of the regimen and baseline liver function may have influenced these results.

This study is limited by its analysis of only a Japanese cohort and potential treatment selection bias in the inclusion of the LE group. Although PSM was performed, asserting that the bias was eliminated is difficult. Nevertheless, selecting the LE group with PS scores up to 2 based on liver reserve function can be considered a valid approach.

While geriatric assessment tools such as sarcopenia and frailty are important indicators of functional status in elderly patients, they could not be adequately evaluated in this cohort, representing a study limitation. Incorporating assessments of general condition, such as the modified G8 score [42] or sarcopenia‐related evaluations [43], could have enabled a more comprehensive analysis. Regarding the comparison by regimen, a rigorous analysis of the DT regimen was limited by the small number of cases and the relatively short observation period; thus, the findings should be interpreted with caution. In addition, because this was a multicenter study, it was challenging to establish standardized inclusion criteria for post‐ICI treatment, which may have introduced variability in treatment selection and outcomes.

### 4.4 Implications and Future Research Directions

This study has significant implications for establishing criteria for immunotherapy in elderly patients with advanced HCC in real‐world clinical practice. Even in elderly patients, initiating treatment at ALBI grade 1 or 2a can lead to a prognosis of approximately 30 months. In addition, given the limited post‐treatment options, the patient's quality of life must be prioritized when making treatment decisions for first‐line immunotherapy. In the non‐LE group, post‐ICI treatment significantly improved OS, particularly among patients receiving locoregional therapies, followed by those treated with systemic therapies (*p* < 0.001, Figure S5).

**FIGURE 4:**
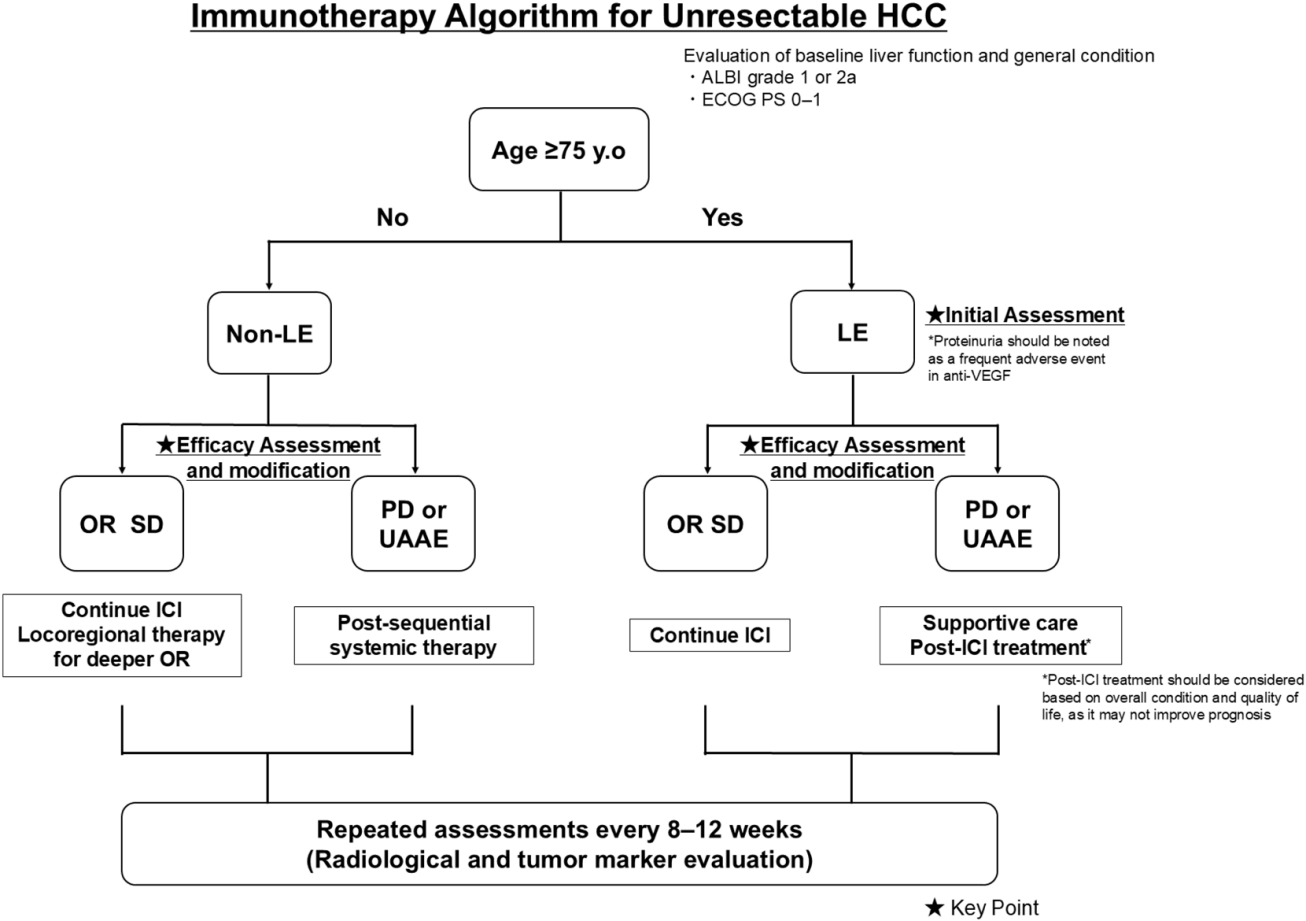
Immunotherapy algorithm for unresectable hepatocellular carcinoma, stratified by age and liver function. ALBI, albumin‐bilirubin grade; anti‐VEGF, anti‐vascular endothelial growth factor; ECOG PS, eastern cooperative oncology group performance status; HCC, hepatocellular carcinoma; ICI, immune checkpoint inhibitor; LE, late elderly; Non‐LE, non‐late elderly; OR, objective response; PD, progressive disease; QOL, quality of life; SD, stable disease; UAAE, unacceptable adverse event.

A recent systematic review and meta‐analysis reported better access to curative treatments and superior survival in younger patients, supporting our findings of age‐related disparities [44]. The limited post‐ICI treatment in the LE group may reflect clinical constraints or under‐recognition of potential candidates. Importantly, the current initial assessment based on liver function for ICI administration appears appropriate for LE patients. However, better strategies are needed to identify which LE patients may benefit from post‐treatment interventions, warranting further research. Therefore, careful monitoring of imaging evaluations and serum data is important during treatment. The findings of this study provide a strategy for a world facing increasing aging populations and advancements in anti‐HCC therapies. A treatment algorithm for elderly patients with unresectable hepatocellular carcinoma was proposed (Figure 4).

In conclusion, the clinical significance of immunotherapy in LE patients with unresectable HCC was highlighted. This demonstrates that initiating first‐line immunotherapy in ALBI grade 1 or 2a subgroups can result in favorable survival outcomes, even in the absence of subsequent post‐ICI treatments, while maintaining a manageable safety profile. In non‐LE patients, the continuation of sequential therapy, incorporating both systemic and locoregional approaches, contributes to improved prognosis. Immunotherapy for HCC in elderly patients should consider liver function and overall condition. Evaluating the ALBI score can lead to improved prognosis of elderly patients.

## Author Contributions

**Shun Kaneko:** conceptualization (lead), data curation (equal), formal analysis (equal), funding acquisition (equal), methodology (equal), project administration (equal), resources (equal), writing – original draft (equal). **Yasuhiro Asahina:** conceptualization (equal), funding acquisition (equal), methodology (equal), project administration (lead), resources (equal), writing – review and editing (equal). **Miyako Murakawa:** data curation (equal), formal analysis (supporting), funding acquisition (equal), methodology (supporting), resources (equal), visualization (supporting). **Shunsuke Ueyama:** data curation (equal), validation (supporting). **Hideki Watanabe:** data curation (equal). **Chiaki Maeyashiki:** data curation (equal). **Akiko Kusano‐Kitazume:** data curation (equal). **Ayako Sato:** data curation (equal). **Kozue Uchidate:** data curation (equal). **Taro Watabe:** data curation (equal). **Takehito Asakawa:** data curation (equal). **Sho Watanabe:** data curation (equal). **Junko Fujiki:** data curation (equal). **Isamu Shibata:** data curation (equal). **Shinya Oooka:** data curation (equal). **Hitoshi Kurata:** data curation (equal). **Mao Tsuchiya:** data curation (equal). **Takashi Fujii:** data curation (equal). **Keiichi Akahoshi:** data curation (equal), validation (supporting), writing – review and editing (supporting). **Daisuke Ban:** supervision (supporting). **Kento Inada:** data curation (equal). **Tsubasa Nobusawa:** data curation (equal). **Tomohiro Mochida:** data curation (equal). **Keiya Watakabe:** data curation (equal). **Taro Shimizu:** data curation (equal). **Jun Tsuchiya:** data curation (equal). **Masato Miyoshi:** funding acquisition (equal), resources (equal), supervision (supporting), visualization (supporting). **Fukiko Kawai‐Kitahata:** data curation (equal), funding acquisition (equal), resources (equal). **Sayuri Nitta:** data curation (equal), funding acquisition (equal), resources (equal). **Mina Nakagawa:** funding acquisition (equal), resources (equal), validation (supporting). **Sei Kakinuma:** funding acquisition (equal), resources (equal), supervision (supporting). **Ryuichi Okamoto:** conceptualization (equal), funding acquisition (equal), supervision (lead), writing – review and editing (equal).

## Ethics Statement

The ethics committee of the Institute of Science Tokyo Hospital approved this study, which was conducted in accordance with the 1913 revision of the Declaration of Helsinki (confirmation number: M2018‐223).

## Consent

This was not applicable; with information made public on the website of the university, the participants were allowed to opt‐out of this study.

## Conflicts of Interest

The authors declare no conflicts of interest.

## Supporting information


**Figure S1:** Overall survival by treatment regimen in the non‐LE and LE groups.
**Figure S2:** Overall survival stratified by prior treatment history and tyrosine kinase inhibitor use in non‐LE and LE groups.
**Figure S3:** Overall survival adjusted by propensity score‐matched ALBI score and AFP. (A) Overall survival by age group. (B) Overall survival stratified by post‐ICI treatment and age group.
**Figure S4:** Association of UPCR and qualitative proteinuria, and prognosis.
A. Correlation between UPCR and qualitative proteinuria.
B. Overall survival based on qualitative proteinuria (graded as 0, ±, 1+ to 4+) or ≥ 2+.
**Figure S5:** Overall survival in the non‐LE and LE groups according to the presence or absence of post‐ICI treatment (locoregional or systemic therapy).

**Data S2:** cam471171‐sup‐0002‐DataS1.docx.

## Data Availability

The data that support the findings of this study are available from the corresponding author upon reasonable request.
