# Supplementary material for: Effect of Immunotherapy on Late Elderly Patients With Unresectable Hepatocellular Carcinoma: A Real‐World Clinical Study
Source: Cancer Med. 2025 Aug 26;14(17):e71171. doi: 10.1002/cam4.71171 (PMC12378699; doi:10.1002/cam4.71171)
Supplement: Supplementary file 1 — Figure S1: Overall survival by treatment regimen in the non‐LE and LE groups. Figure S2: Overall survival stratified by prior treatment history and tyrosine kinase inhibitor use in non‐LE and LE groups. Figure S3: Overall survival adjusted by propensity score‐matched ALBI score and AFP. (A) Overall survival by age group. (B) Overall survival stratified by post‐ICI treatment and age group. Figure S4: Association of UPCR and qualitative proteinuria, and prognosis. A. Correlation between UPCR and qualitative proteinuria. B. Overall survival based on qualitative proteinuria (graded as 0, ±, 1+ to 4+) or ≥ 2+. Figure S5: Overall survival in the non‐LE and LE groups according to the presence or absence of post‐ICI treatment (locoregional or systemic therapy). [file CAM4-14-e71171-s001.pptx]

## Slide 1
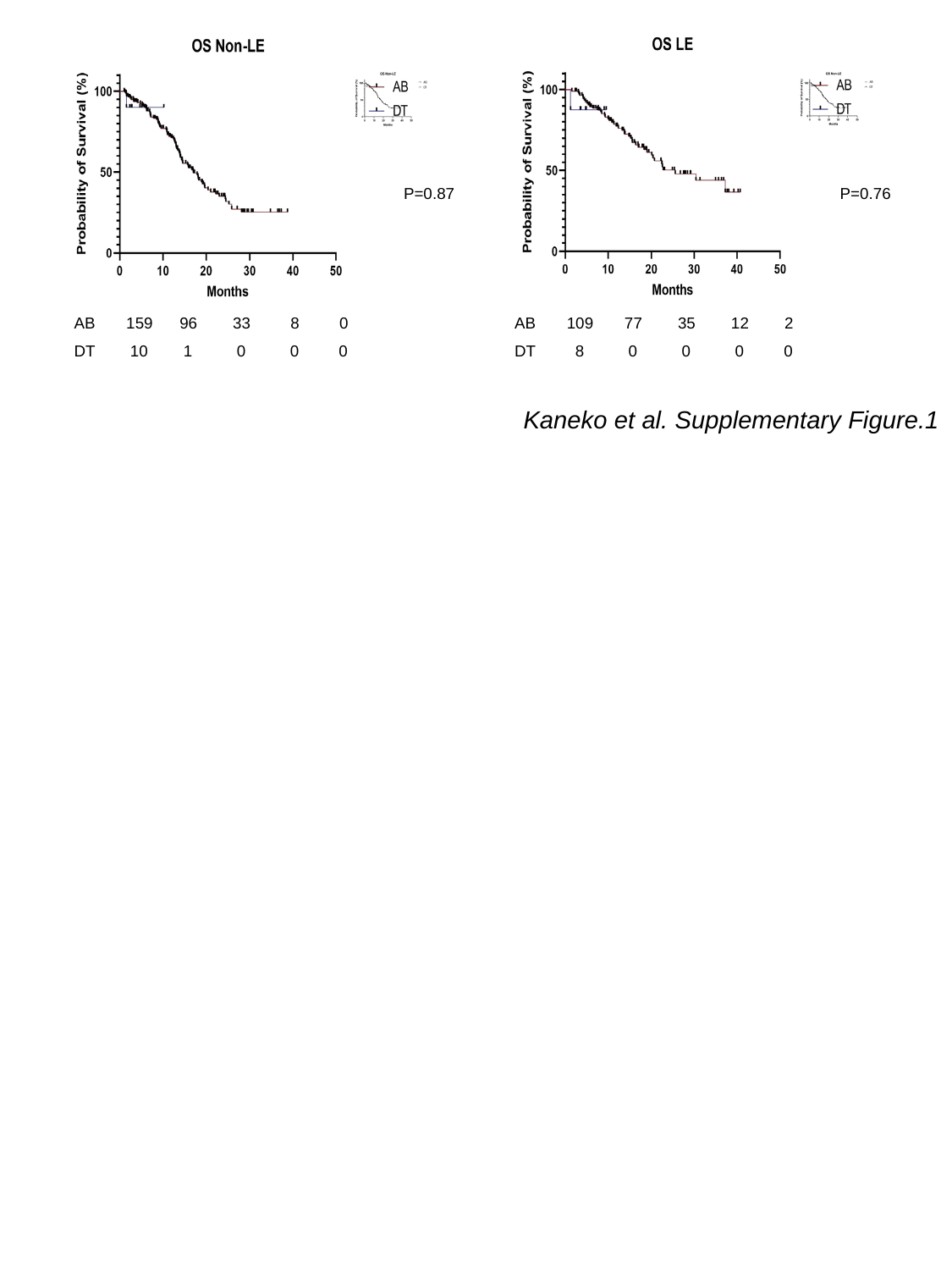

P=0.87
P=0.76
AB 159 96 33 8 0
DT 10 1 0 0 0
AB 109 77 35 12 2
DT 8 0 0 0 0
Kaneko et al. Supplementary Figure.1

## Slide 2
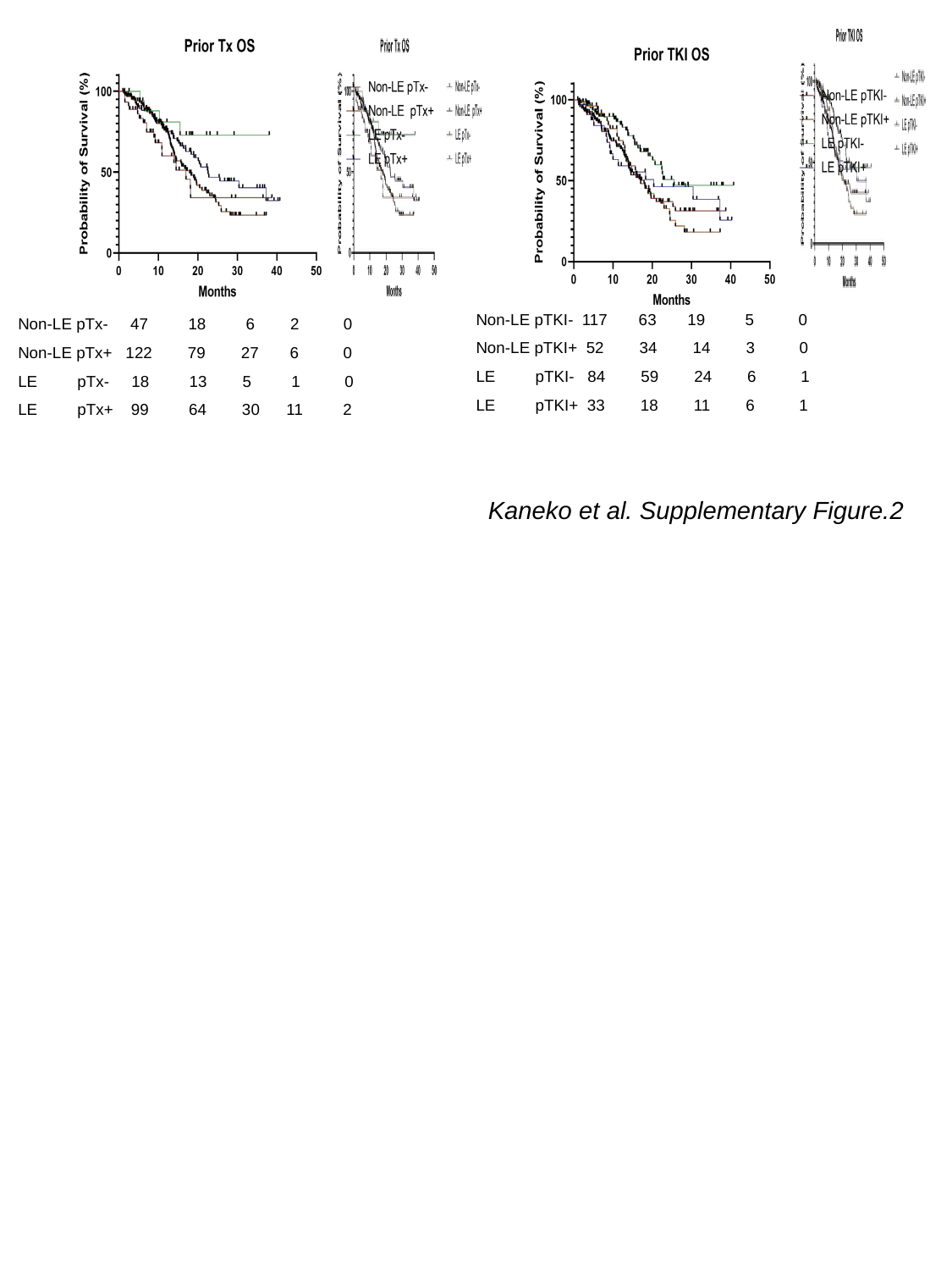

Non-LE pTKI- 117 63 19 5 0
Non-LE pTKI+ 52 34 14 3 0
LE pTKI- 84 59 24 6 1
LE pTKI+ 33 18 11 6 1
Non-LE pTx- 47 18 6 2 0
Non-LE pTx+ 122 79 27 6 0
LE pTx- 18 13 5 1 0
LE pTx+ 99 64 30 11 2
Kaneko et al. Supplementary Figure.2

## Slide 3
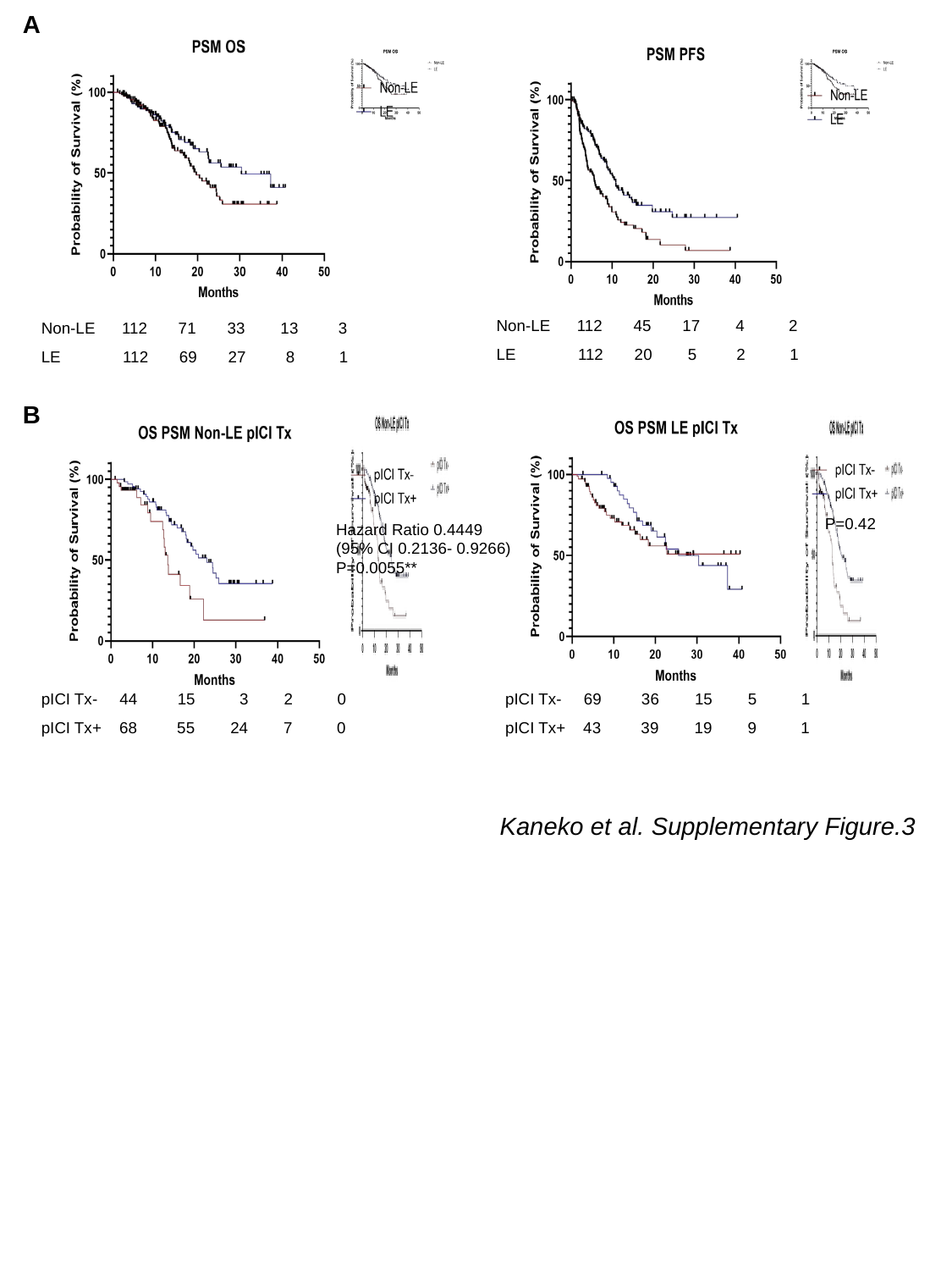

A
Non-LE 112 45 17 4 2
LE 112 20 5 2 1
Non-LE 112 71 33 13 3
LE 112 69 27 8 1
B
P=0.42
Hazard Ratio 0.4449
(95% CI 0.2136- 0.9266)
P=0.0055**
pICI Tx- 44 15 3 2 0
pICI Tx+ 68 55 24 7 0
pICI Tx- 69 36 15 5 1
pICI Tx+ 43 39 19 9 1
Kaneko et al. Supplementary Figure.3

## Slide 4
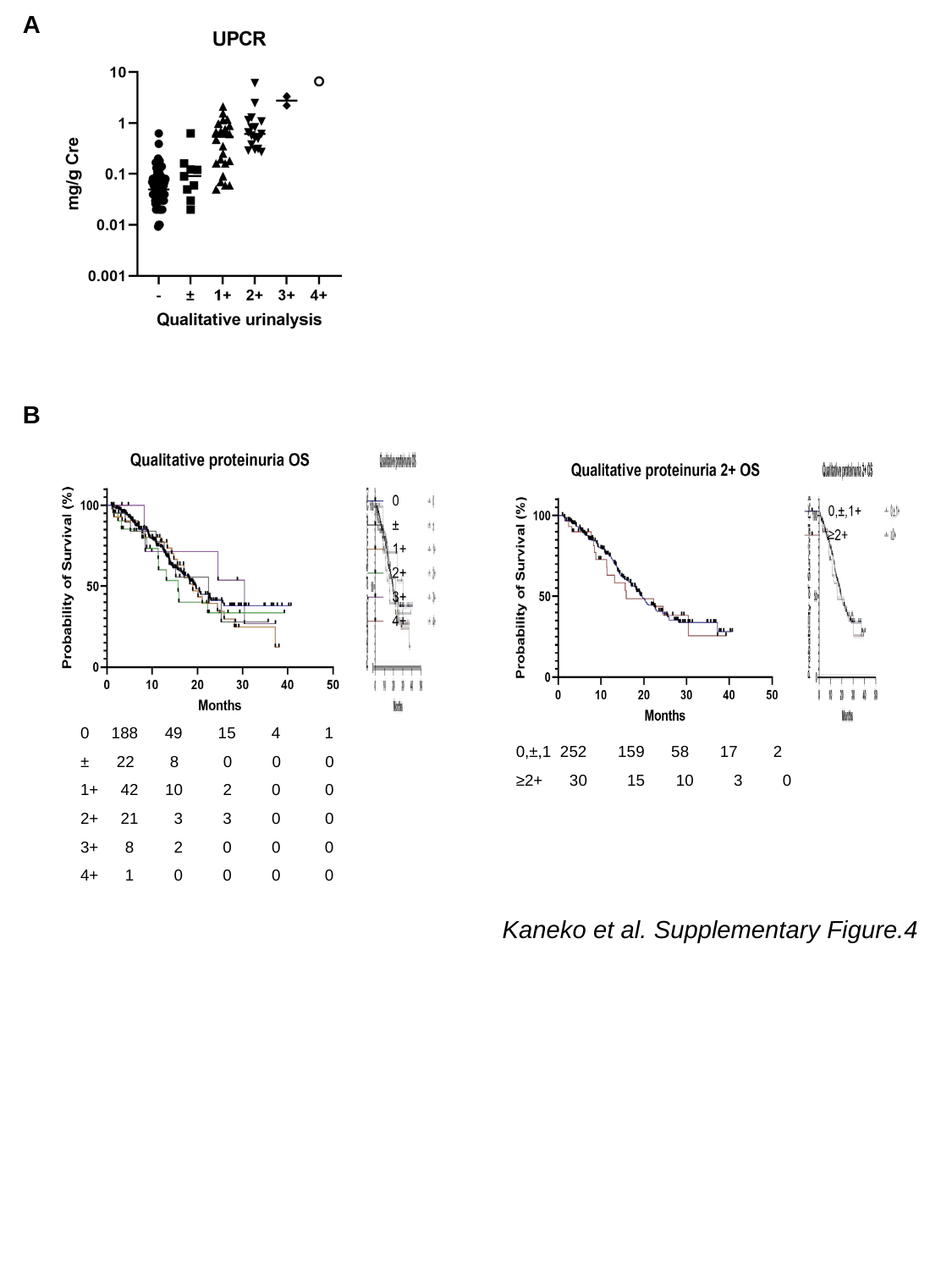

A
B
0 188 49 15 4 1
±　 22 8 0 0 0
1+ 42 10 2 0 0
2+ 21 3 3 0 0
3+ 8 2 0 0 0
4+ 1 0 0 0 0
0,±,1 252 159 58 17 2
≥2+ 30 15 10 3 0
Kaneko et al. Supplementary Figure.4

## Slide 5
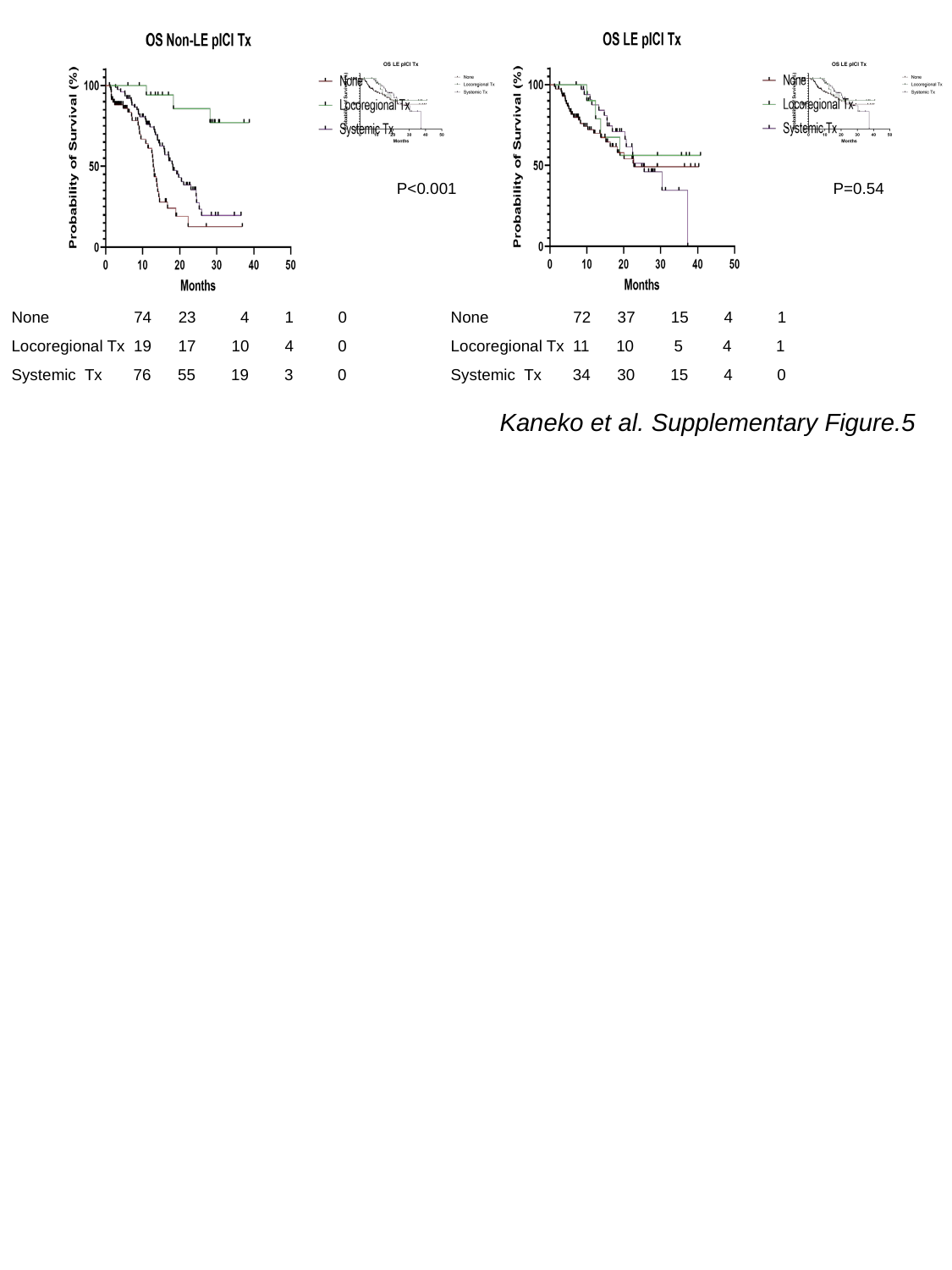

P<0.001
P=0.54
None 74 23 4 1 0
Locoregional Tx 19 17 10 4 0
Systemic Tx 76 55 19 3 0
None 72 37 15 4 1
Locoregional Tx 11 10 5 4 1
Systemic Tx 34 30 15 4 0
Kaneko et al. Supplementary Figure.5
