# Supplementary material for: Effect of Immunotherapy on Late Elderly Patients With Unresectable Hepatocellular Carcinoma: A Real‐World Clinical Study
Source: Cancer Med. 2025 Aug 26;14(17):e71171. doi: 10.1002/cam4.71171 (PMC12378699; doi:10.1002/cam4.71171)
Supplement: Supplementary file 2 — Data S2: cam471171‐sup‐0002‐DataS1.docx. [file CAM4-14-e71171-s002.docx]

**Supplementary Tables.**

**Supplementary Table 1. clinical characteristics of patients treated with either atezolizumab plus bevacizumab or durvalumab plus tremelimumab**

**A: Baseline clinical characteristics**

|  | AB | DT | P value |
| --- | --- | --- | --- |
| n | 268 | 18 |  |
| Age (years), median (range) | 73 (34-91) | 73.5 (47-82) | 0.799 |
| Sex：Male (n, %) | 230 (85.8%) | 17 (94.4%) | 0.483 |
| BMI | 23.38 (15.4- 41.6) | 22.41 (18.14-35.48) | 0.608 |
| ECOG PS 0/1/2 | 210/54/4 | 15/3/0 | 0.999 |
| Etiology (viral/non-viral) |  |  | 0.143 |
| HBV | 37 (13.8%) | 1 (5.6%) |  |
| HCV | 71 (26.5%) | 3 (16.7%) |  |
| Alcohol | 75 (28.0%) | 8 (44.4%) |  |
| Others | 85 (31.7%) | 6 (34.4%) |  |
| Child-Pugh Class |  |  | 0.999 |
| A | 224 (83.6%) | 15 (83.3%) |  |
| B | 44 (16.4%) | 3 (16.7%) |  |
| ALBI score | -2.42 (-3.38- -0.40) | -2.23 (-3.10- -1.57) | 0.541 |
| mALBI grade 1/2a/2b/3 | 87/75/96/10 | 5/4/9/0 | 0.753 |
| mALBI grade 1/2a | 162 (60.4%) | 9 (50.0%) | 0.459 |
| AFP (ng/mL) | 24.3 (1.0-852122) | 10.1 (1.5-8042) | 0.125 |
| AFP>100 (ng/mL) | 105 (39.2%) | 3 (16.7%) | 0.077 |
| NLR | 2.58 (0.19-118.75) | 2.68 (1.46-8.16) | 0.993 |
| Cirrhosis (n, %) | 190 (70.9%) | 14 (77.8%) | 0.788 |
| ALT (IU/L) | 27 (6-202) | 28 (6-83) | 0.891 |
| Creatinine (mg/dL) | 0.81 (0.32-5.43) | 0.93 (0.59-1.64) | 0.321 |
| eGFR (ml/min/1.73m^2^) | 68.55 (8.9-168.9) | 62.3 (32.1-104.3) | 0.424 |
| CRP (mg/dL) | 0.28 (0.01-12.8) | 0.22 (0.02-3.26) | 0.432 |
| BCLC stage |  |  | 0.506 |
| 0/A (early stage) | 23 (8.6%) | 2 (11.1%) |  |
| B (intermediate stage) | 146 (54.5%) | 12 (66.7%) |  |
| C (advanced stage) | 97 (36.2%) | 4 (22.2%) |  |
| Treatment line 1st/2nd/3rd/4th/5th/6th | 185/58/12/9/2/2 | 14/3/1/0/0/0 | 0.924 |
| LE/non-LE | 109 (40.7%) | 8 (44.4%) | 0.807 |

1. **Best radiological response assessed RECIST 1.1**

| AB | Non-LE (≤74 y.o) | LE  (≥75 y.o) | P value |
| --- | --- | --- | --- |
| CR (n, %) | 5 (3.1%) | 10 (9.2%) |  |
| PR (n, %) | 31 (19.5%) | 26 (23.9%) |  |
| SD (n, %) | 74 (46.6%) | 54 (49.5%) |  |
| PD (n, %) | 49 (30.8%) | 19 (17.4%) |  |
| ORR (%) | 22.6% | 32.1% | 0.0687 |
| DCR (%) | 69.2% | 82.6% | 0.0151* |

| DT | Non-LE (≤74 y.o) | LE  (≥75 y.o) | P value |
| --- | --- | --- | --- |
| CR (n, %) | 0 (0.0%) | 0 (0.0%) |  |
| PR (n, %) | 3 (30.0%) | 2 (25.0%) |  |
| SD (n, %) | 5 (50.0%) | 4 (25.0%) |  |
| PD (n, %) | 2 (20.0%) | 2 (25.0%) |  |
| ORR (%) | 30.0% | 25.0% | 0.999 |
| DCR (%) | 80.0% | 75.0% | 0.999 |

AB, atezolizumab and bevacizumab; DT, durvalumab + tremelimumab

LE, late elderly;

CR, complete response; PR, partial response; SD, stable disease;

PD, progressive disease; ORR, objective response rate; DCR, disease control rate.

**Supplementary Table 2. Breakdown of Non–Liver-Related Causes of Death**

|  | Non-LE (≤74 y.o) | LE (≥75 y.o) |
| --- | --- | --- |
| Sepsis | 3 |  |
| Cerebral Hemorrhage | 2 |  |
| Cerebral Infarction | 1 |  |
| Unknown | 2 | 2 |
| COVID19 |  | 1 |
| Gastrointestinal Bleeding |  | 1 |
| Myelodysplastic Syndrome |  | 1 |
| Tongue Cancer |  | 1 |
| Heart Failure |  | 1 |
| Myasthenia Gravis |  | 1 |

**Supplementary Table.3 The breakdown of post ICI treatment in Non-LE and LE patients**

|  | Non LE (≤74 y.o) | LE (≥75 y.o) |
| --- | --- | --- |
| n | 95 | 45 |
| LEN | 53 (55.8%) | 25 (55.6%) |
| CAB | 13 (13.7%) | 1 (2.2%) |
| TACE | 11 (11.6%) | 7 (15.6%) |
| SOR | 3 (3.2%) | 4 (8.9%) |
| RAM | 3 (3.2%) | 0 (0.0%) |
| RFA | 3 (3.2%) | 2 (4.4%) |
| Surgery | 3 (3.2%) | 0 (0.0%) |
| REG | 2 (2.1%) | 1 (2.2%) |
| DT | 1 (1.1%) | 2 (4.4%) |
| RT | 1 (1.1%) | 2 (4.4%) |
| D | 0 (0.0%) | 1 (2.2%) |
| HAIC | 1 (1.1%) | 0 (0.0%) |
| Other | 1 (1.1%) | 0 (0.0%) |

LEN, lenvatinib; CAB, cabozantinib; TACE, transcatheter arterial chemoembolization;

SOR, sorafenib; RAM, ramcirumab; RFA, radiofrequency ablation;

REG, regorafenib; DT, durvalumab + tremelimumab; RT, radiation therapy:

D, durvalumab; HAIC, hepatic arterial infusion chemotherapy

**Supplementary Table.4 Baseline clinical characteristics adjusted by propensity score matched with ALBI score and AFP**

|  | Non-LE (≤74 y.o) | LE (≥75 y.o) | P value |
| --- | --- | --- | --- |
| n | 112 | 112 |  |
| Age (years), median (range) | 68 (34-74) | 79 (75-91) | <0.001*** |
| Sex：Male (n, %) | 97 (86.6%) | 93 (83.0%) | 0.999 |
| BMI | 22.9 (15.4- 41.6) | 23.5 (16.0-36.3) | 0.163 |
| ECOG PS 0/1/2 | 96/15/1 | 78/31/3 | 0.01* |
| Etiology (viral/non-viral) |  |  | 0.17 |
| HBV | 22 (19.6%) | 8 (7.1%) |  |
| HCV | 27 (24.1%) | 30 (26.8%) |  |
| Alcohol | 33 (29.5%) | 29 (25.9%) |  |
| Others | 30 (26.8%) | 45 (40.1%) |  |
| Child-Pugh Class |  |  | 0.218 |
| A | 95 (84.8%) | 102 (91.1%) |  |
| B | 17 (15.2%) | 10 (8.9%) |  |
| ALBI score | -2.45 (-3.36- -1.28) | -2.48 (-3.38- -1.27) | 0.874 |
| mALBI grade 1/2a/2b/3 | 39/38/32/3 | 40/37/33/2 | 0.999 |
| AFP (ng/mL) | 25.98 (1.5-28763) | 14.6 (1.0-375007) | 0.082 |
| NLR | 2.55 (0.7-11.1) | 2.56 (0.19-8.01) | 0.957 |
| Cirrhosis (n, %) | 78 (69.6%) | 75 (67.0%) | 0.774 |
| ALT (IU/L) | 27 (9-165) | 23 (6-158) | 0.015* |
| Creatinine (mg/dL) | 0.78 (0.32-2.07) | 0.84 (0.45-1.93) | 0.131 |
| CRP (mg/dL) | 0.26 (0.02-10.7) | 0.27 (0.01-9.13) | 0.556 |
| BCLC stage |  |  | 0.269 |
| 0/A (early stage) | 10 (8.9%) | 13 (11.6%) |  |
| B (intermediate stage) | 58 (51.8%) | 68 (60.7%) |  |
| C (advanced stage) | 44 (39.3%) | 31 (27.7%) |  |
| Treatment line 1st/2nd/3rd/4th/5th/6th | 79/27/2/2/1/1 | 81/18/6/6/0/1 | 0.182 |
| AB or DT | 104/8 | 105/7 | 0.999 |

LE, late elderly;

BMI, body mass index; PS, performance status; HBV, hepatitis B virus; HCV, hepatitis C virus;

ALBI, albumin-bilirubin; mALBI, modified ALBI; AFP, Alpha fetoprotein;

NLR, neutrophil-to-lymphocyte ratio; ALT, alanine aminotransferase; CRP, C-reactive protein;

BCLC, Barcelona Clinic Liver Cancer; TKI, tyrosine kinase inhibitor;

AB, atezolizumab and bevacizumab; DT, durvalumab + tremelimumab
